# Construction of a Robust Cofactor Self-Sufficient Bienzyme Biocatalytic System for Dye Decolorization and its Mathematical Modeling

**DOI:** 10.3390/ijms20236104

**Published:** 2019-12-03

**Authors:** Haitao Ding, Wei Luo, Yong Yu, Bo Chen

**Affiliations:** MNR Key Laboratory for Polar Science, Polar Research Institute of China, Shanghai 200136, China; luowei@pric.org.cn (W.L.); yuyong@pric.org.cn (Y.Y.); chenbo@pric.org.cn (B.C.)

**Keywords:** glucose 1-dehydrogenase, triphenylmethane reductase, cofactor regeneration, dye decolorization, multiple linear regression, random forest, artificial neural network, modeling

## Abstract

A triphenylmethane reductase derived from *Citrobacter* sp. KCTC 18061P was coupled with a glucose 1-dehydrogenase from *Bacillus* sp. ZJ to construct a cofactor self-sufficient bienzyme biocatalytic system for dye decolorization. Fed-batch experiments showed that the system is robust to maintain its activity after 15 cycles without the addition of any expensive exogenous NADH. Subsequently, three different machine learning approaches, including multiple linear regression (MLR), random forest (RF), and artificial neural network (ANN), were employed to explore the response of decolorization efficiency to the variables of the bienzyme system. Statistical parameters of these models suggested that a three-layered ANN model with six hidden neurons was capable of predicting the dye decolorization efficiency with the best accuracy, compared with the models constructed by MLR and RF. Weights analysis of the ANN model showed that the ratio between two enzymes appeared to be the most influential factor, with a relative importance of 54.99% during the decolorization process. The modeling results confirmed that the neural networks could effectively reproduce experimental data and predict the behavior of the decolorization process, especially for complex systems containing multienzymes.

## 1. Introduction

Triphenylmethane dyes such as malachite green and crystal violet are extensively applied in the textile industry for dyeing [1] and in the aquaculture industry as antifungal agents [2]. However, concerns have been raised about the severe environmental and health impacts of the industrial effluent discharges containing these recalcitrant dyes, which are generally regarded as toxic, mutagenic, and carcinogenic [3]. Consequently, a variety of biotreatment methods for triphenylmethane dye removal have been developed based on microbes or enzymes [4,5,6].

A specific enzyme, triphenylmethane reductase (TMR), was first discovered in *Citrobacter* sp. KCTC 18061P (CsTMR), and is capable of catalyzing the decolorization of triphenylmethane dyes to their leuco-derivatives using NAD(P)H as cofactors [7]. On account of its high activity and considerable stability, CsTMR provides a promising alternative for the biological removal of triphenylmethane dyes. However, the practical application of this enzyme is limited by its indispensable requirement for the costly cofactor, as do other nicotinamide coenzyme-dependent oxidoreductases [8]. To cope with the dilemma, different coenzyme regeneration systems have been proposed to continuously provide cofactors such as NAD(P)/NAD(P)H in vitro [9]. As one of the enzymes widely used for cofactor regeneration, glucose 1-dehydrogenase (GDH), which catalyzes the oxidation of β-D-glucose to produce D-glucono-1,5-lactone, converting NAD(P) to NAD(P)H concomitantly, has the advantages of dual cofactor specificity and high activity over other cofactor regeneration enzymes [10,11]. Therefore, GDH can be coupled with TMR to construct a self-sufficient system for the decolorization of triphenylmethane dyes.

Although numerous multienzyme systems have been broadly applied in biosensors [12], biosynthesis [13], pharmaceutical manufacturing [14], etc., it is still difficult to analyze system behavior and recognize the influential variables involved in these complex systems. To the best of our knowledge, no existing mathematical model can be directly applied to describe the kinetics of a multienzyme system with key parameters that affect the catalytic efficiency significantly. Since the nonlinear kinetic behavior of these systems cannot be simply modeled by traditional models such as the Michaelis–Menten equation and its derivatives, it is necessary to employ powerful tools to solve such problems. Beyond the ordinary rule-based algorithms, artificial neural networks (ANNs), inspired by biological neural networks, have been proven to be a robust modeling tool able to solve a wide variety of highly nonlinear tasks [15], including prediction, optimization, troubleshooting, computer vision, speech recognition, etc. In addition to ANNs, the random forest (RF) algorithm proposed by Leo Breiman [16], which can deal with complex structures as well as highly correlated variables with excellent performance [17], has also become another popular machine learning tool in both scientific and industrial communities in recent years.

**Figure 1:**
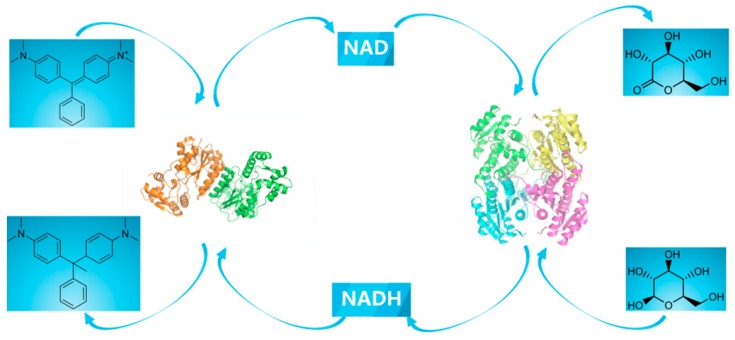
Scheme of the bienzyme dye decolorization system constructed in this study.

In the present work, a thermal-stable GDH [18] from *Bacillus* sp. ZJ (BzGDH) was coupled with CsTMR to construct an efficient bienzyme system able to catalyze the reversible interconversion of NAD and NADH simultaneously with the decolorization of malachite green to leucomalachite green (Figure 1). Three machine learning algorithms, including multiple linear regression (MLR), random forest (RF), and artificial neural network (ANN), were implemented to model the decolorization behavior fulfilled by the self-sufficient bienzyme dye decolorization system.

## 2. Results and Discussion

### 2.1. Construction of a Self-Sufficient Bienzyme Biocatalytic System for Dye Decolorization

**Figure 2:**
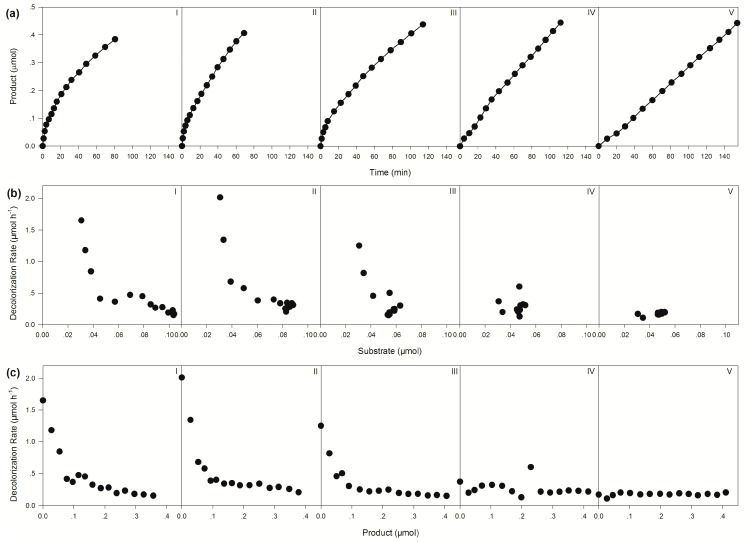
Performance of the self-sufficient bienzyme biocatalytic system for dye decolorization. (**a**) Changes in product yield over time. (**b**) Relationship between the amount of substrate and decolorization rate. (**c**) Relationship between the amount of product and decolorization rate. Molar ratios between CsTMR and BzGDH were set as 1:1 (I), 1:5 (II), 1:10 (III), 5:1 (IV), and 10:1 (V).

A self-sufficient bienzyme biocatalytic system composed of BzGDH, CsTMR, NAD, and glucose was constructed for dye decolorization. Figure 2a shows the performance of the batch trials conducted using the different molar ratios of BzGDH and CsTMR, suggesting that this biocatalytic system could be efficiently applied in dye removal and maintain its activity after 15 batches, without the addition of any expensive exogenous NADH. As shown in Table 1, the molar ratio of 1:5 for CsTMR/BzGDH displayed the highest initial and average decolorization rate; either increase or decrease in the proportion of CsTMR caused a decrease in decolorization efficiency, indicating that CsTMR should be in proper ratio with BzGDH in the system to achieve a high dye degradation efficiency.

It is worth to point out that the bienzyme catalytic system showed obvious product inhibition, especially when a high proportion of BzGDH was involved (Figure 2b,c). A reasonable explanation for this phenomenon is that a higher proportion of BzGDH provides more NADH for CsTMR at the initial stage, which results in a fast accumulation of products. Consequently, the accumulated product competes with the substrate for the active sites of CsTMR, resulting in product inhibition as slowing down the decolorization efficiency significantly.

### 2.2. Modeling by Multiple Linear Regression

In general, the multiple linear regression method has been often applied to exploring the linear relationship between independent variables and dependent variables. In this study, the corresponding linear model obtained using the entire dataset with three input variables was as follows:

*v* = 0.9833 + 0.0707 [*Ratio*] − 12.7124 [*Substrate*] − 0.5348 [*Product*] (1)

This linear model was then utilized to predict the dye decolorization efficiency. The value of correlation coefficient R^2^ was calculated as 0.5421 for the best fit of experimental versus predicted values, with an equation of *y* = 0.6022 *x* + 0.1616, indicating a weak linear relationship between dye decolorization efficiency and three independent variables, including molar ratio of CsTMR/BzGDH, concentration of substrate, and concentration of product.

### 2.3. Modeling by Random Forest

**Figure 3:**
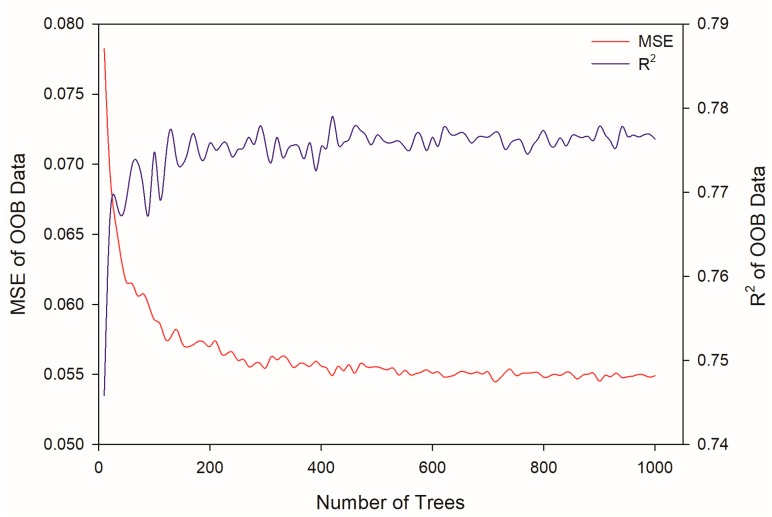
Mean square error (MSE) and correlation coefficient (R^2^) of models trained by random forest with the different number of trees. OOB: out-of-bag.

To determine the best tree number to be adopted in the modeling stage, all samples were modeled using the random forest algorithm with different numbers of trees, which were set from 10 to 1000 with intervals of 10. Mean square error (MSE) and correlation coefficient (R^2^) were used as criteria to determine the optimum numbers of trees. According to Figure 3, both the MSE and R^2^ converged when the tree numbers were more than 600. Therefore, the tree number was set to 600 to train the final model on the training subset with minimized MSE of OOB (out-of-bag) data. The model was subsequently used to predict the dye decolorization efficiency; the best fit was described by the equation *y* = 1.4602 *x* − 0.1573, with an R^2^ of 0.7423.

### 2.4. Modeling by Artificial Neural Network

**Figure 4:**
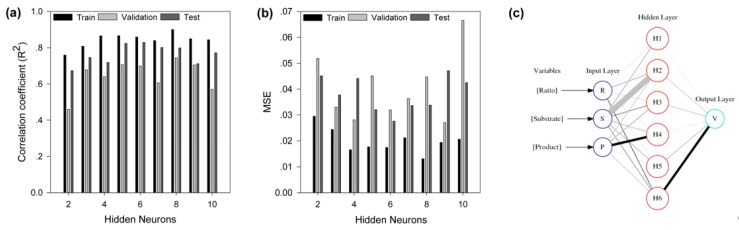
Training, validation, test, and interpretation of the neural network. (**a**) The correlation coefficient (R^2^) of the trained back-propagation artificial neural network (BP-ANN) with different hidden neurons. (**b**) Mean square error (MSE) of the trained BP-ANN with different hidden neurons. (**c**) Neural interpretation diagram (NID) of the network. The shade of the lines connecting neurons indicates the direction of the interaction between them, and the black connection is positive (activator) and the grey connection is negative (inhibitor). The thickness of the lines is scaled to the magnitude of the connection weights between neurons.

Since ANN has been proven to be a robust strategy to unravel the relationships among variables, especially for non-linear relationships, we adopted BP-ANN in this study to explore the response of decolorization efficiency to the dependent variables of the bienzyme biocatalytic system. The primary goal of network training was to minimize the error function MSE by searching for a weight matrix that could reproduce the predicted outputs as equal or close to the experimental values. To avoid over-fitting, the optimum number of hidden nodes was determined by 10-fold cross-validation technique. Both the MSE and R^2^ between the predicted and experimental values of the training, validation and test subsets suggested that network with six neurons in the hidden layer had the best performance, with over-fitting avoided (Figure 4a,b). Therefore, a three-layer feed-forward back-propagation neural network with six hidden neurons, represented by a neural interpretation diagram (Figure 4c), was employed to model the decolorization process implemented by the bienzyme system.

### 2.5. Model Comparision

**Figure 5:**
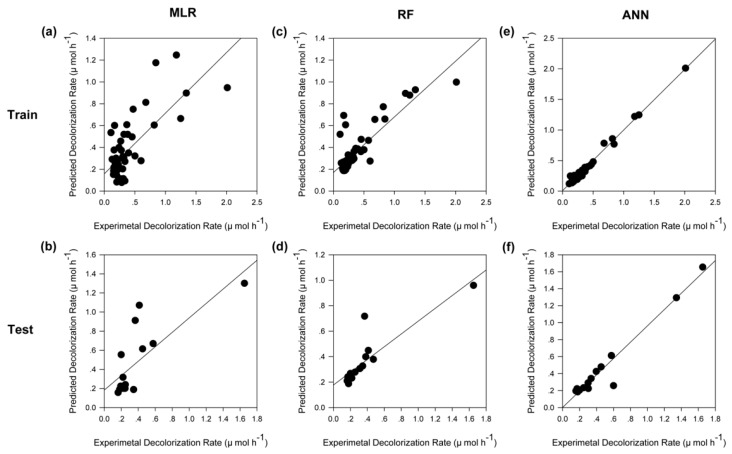
Comparisons between experimental and predicted values of different models. (**a**,**c**,**e**) The constructed MLR, RF, and ANN models applied to the dataset used for training, respectively. (**b**,**d**,**f**) The constructed MLR, RF, and ANN models applied to the dataset used for testing, respectively. The line represents the best fit of the scatter plot, which was obtained by regression analysis based on minimization of the squared errors.

Generally, a good model should have considerable generalization capability; to evaluate the generalization capability of a model fairly, the test dataset should be independent of the training dataset. As indicated in Section 3, the entire dataset was used only to select the hyperparameters such as tree numbers of RF and hidden neuron number of ANN, to avoid over-fitting. In the model training stage, the entire dataset was randomly divided into two datasets, a training dataset and test dataset. Subsequently, only the training dataset was employed to train models; the test dataset was not involved in this process. Finally, we used the test dataset, which was obtained from experiments, to comprehensively evaluate the generalization capability of the constructed models. Figure 5 illustrates a comparison between experimental values and predicted output values using different models. Of all predictions for the test datasets which were not involved in the construction of models (Figure 5b,d,f), the ANN model behaved the best, with an equation of *y* = 0.9783 *x* + 0.0086 and a correlation coefficient (R^2^) value of 0.9757 (Figure 5f). The results confirmed that the neural network model could effectively reproduce the experimental results.

The accuracy of the prediction of the constructed models was further estimated by different statistical parameters including MSE and R^2^, mean absolute error (MAE, Equation (5)), and mean relative error (MRE, Equation (6)), respectively. These statistical parameters also confirmed the best predictive capability of ANN (Table 2).

### 2.6. Weights Analysis of ANN

The weight between two artificial neurons is analogous to the synapse strength between axon and dendrite in real biological neurons. Consequently, each weight of the neural network determines the percentage of the signal strength of an input neuron that will be transmitted to the output neuron. The neural network weight matrix (Table 3) can be used to estimate the relative importance of the various input variables on the output variables. The relative importance of input variables was estimated by different approaches (Table 4). As indicated in Section 3.1, the original Garson’s algorithm [19] has two obvious drawbacks: it omits the weight between hidden and output layers, and employs the absolute value of weights for calculation, which would result in erroneous estimation of the contribution of the input variables. Hence, some other weight analysis methods have been proposed to estimate the contribution of variables more accurate. A modified Garson’s algorithm [20] takes the weight linking hidden and output neurons into consideration, while the CWA (connection weight approach) algorithm [21] adopts the raw connection weight rather than the absolute values for calculation. Both the modified Garson’s algorithm and CWA algorithm gave the same ranking of the effects of variables on dye decolorization efficiency. As may be perceived, the ratio of the two enzymes appeared to be the most influential factor, with a relative importance of 54.99% computed by the modified Garson’s algorithm, during the decolorization process (Table 4).

### 2.7. Sensitivity Analysis of ANN

**Figure 6:**
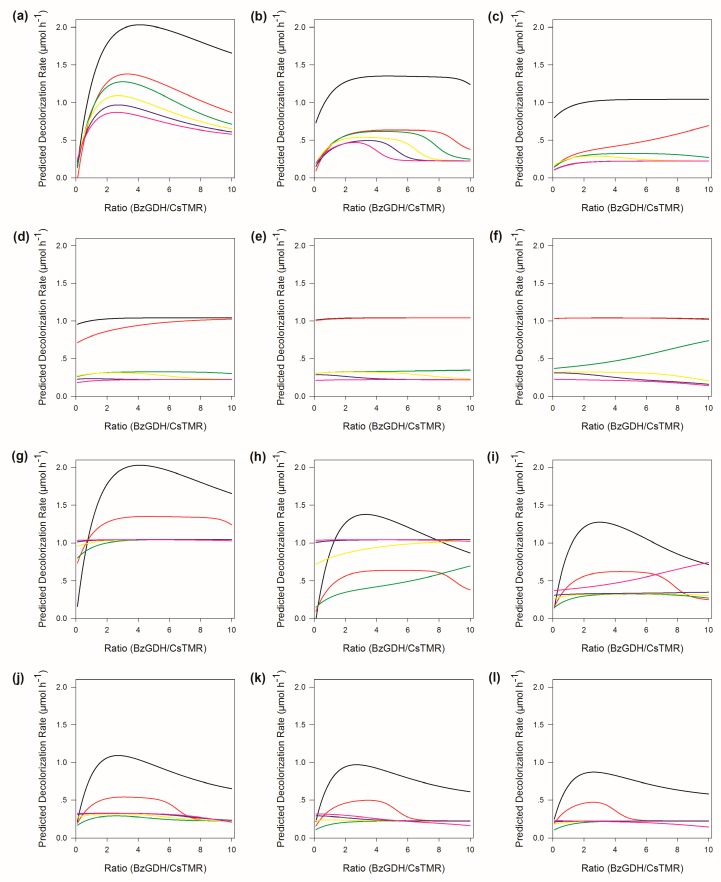
Sensitivity analysis for the variables of modeled neural networks. (**a**–**f**) Response of decolorization rate to changes in the ratio between BzGDH and CsTMR with the substrate held at its 0th, 20th, 40th, 60th, 80th, and 100th percentiles, respectively. Product held at its 0th, 20th, 40th, 60th, 80th, and 100th percentiles is represented as a solid line colored black, red, green, yellow, blue, and magenta, respectively. (**g**–**l**) Response of decolorization rate to changes in the ratio between BzGDH and CsTMR with the product held at its 0th, 20th, 40th, 60th, 80th, and 100th percentiles, respectively. Substrate held at its 0th, 20th, 40th, 60th, 80th, and 100th percentiles is represented as a solid line colored black, red, green, yellow, blue, and magenta, respectively.

To determine the response profile of the output variable to the input variables, several types of sensitivity analysis have been proposed [22]. The traditional sensitivity analysis involves varying each input variable across its entire range while holding all other input variables constant to assess the individual contributions of each variable. In the present work, we adopted Lek’s algorithm [23] with some modification, as described in Section 3.11. Diverse kinds of plots including Gaussian, left-skewed, decreasing, and flat response curves are displayed in Figure 6. The decolorization efficiency decreased with increased concentration of the substrate or product under the same ratio of two enzymes, suggesting that the enzymatic decolorization system possessed obvious substrate inhibition as well as product inhibition, which was in accordance with the experimental results (Figure 2).

### 2.8. The Response of the Decolorization Efficiency to Other Variables

**Figure 7:**
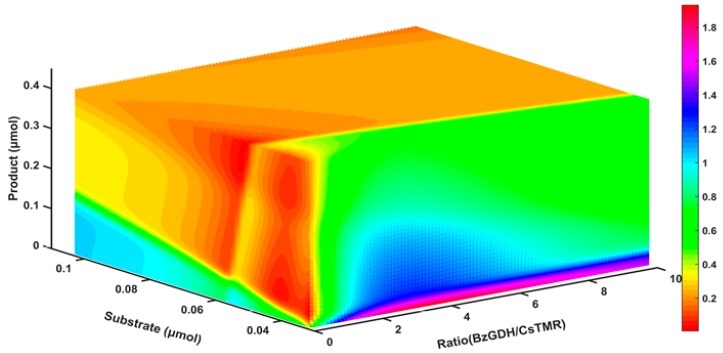
The response of decolorization rate to changes in substrate, product, and bienzyme ratio.

To obtain a panoramic view of the response of the decolorization efficiency to the involved variables including substrate, product, and ratio of the two enzymes, a three-dimensional map was generated by MATLAB. As shown in Figure 7, the bienzyme system achieved its highest decolorization efficiency with a low concentration of substrate and product, and an appropriate bienzyme ratio. With increasing concentration of either substrate or product, the decolorization efficiency reduced sharply, indicating that both substrate and product inhibition could be lead causes of the low decolorization efficiency. Consequently, we propose that a low concentration of dyes is preferred and that the corresponding product should be removed promptly to achieve high decolorization efficiency in practical biological treatment of triphenylmethane dye.

## 3. Materials and Methods

### 3.1. Strains, Plasmids, and Chemicals

The gene encoding for CsTMR derived from *Citrobacter* sp. KCTC 18061P [7] was synthesized and ligated into plasmid pET-28a (+). The plasmid harboring the gene encoding for BzGDH from *Bacillus* sp. ZJ was constructed in our previous study [18]. *E*. *coli* strains DH5α and BL21 (DE3) were used for plasmid amplification and expression, respectively. Malachite green was purchased from Sigma (St. Louis, MO, USA). All other chemicals of analytical grade were purchased from Sangon Biotech (Shanghai, China).

### 3.2. Preparation of Recombinant Enzymes

The recombinant cells were cultured in a 500 mL flask containing 100 mL of Luria-Bertani (LB) medium at 37 °C, with 50 μg/ml of kanamycin added. IPTG (Isopropyl β-d-1-thiogalactopyranoside) of 0.5 mM was added to the medium for induction at 25 °C for 8–12 h when the absorbance at 600 nm of the culture reached 0.5–0.8. The recombinant cells were collected by centrifugation at 10,000× *g* at 4 °C for 10 min, followed by ultrasonic decomposition and nickel-chelating affinity chromatography purification. The eluent was desalted and concentrated with 25 mM sodium phosphate buffer (pH 7.0) containing 10% glycerol using Amicon Ultra Centrifugal Filter (Millipore, Billerica, MA, USA) at 7500× *g* for 30 min. All purification procedures were implemented at 4 °C. The protein concentration was determined by Bradford’s method using bovine serum albumin as the standard.

### 3.3. Enzyme Activity Assays

The activity of BzGDH was determined by measuring the OD_340_ of NADH in 100 mM of phosphate buffer (pH 8.0) with 200 mM of glucose and 1 mM of NAD contained. One unit of BzGDH activity was defined as the amount of the enzyme required to produce 1 μmol of NADH per minute. The activity of CsTMR was assayed by monitoring the OD_616_ of malachite green in 100 mM of phosphate buffer (pH 7.0) containing 200 μM of NADH and 20 of μM malachite green. One unit of CsTMR activity was defined as the amount of the enzyme required to degrade 1 μmol of malachite green per minute. All measurements were conducted at 25 °C.

### 3.4. Construction of a Cofactor Self-Sufficient Bienzyme Biocatalytic System for Dye Decolorization

Fed-batch experiments were conducted in a 50 mL beaker containing 200 mM of glucose, 1 mM of NAD, and 3 µM of malachite green, with a magnetic stirring apparatus at 25 °C. The molar ratios between CsTMR and BzGDH were set at 1:1, 1:5, 1:10, 5:1, and 10:1. Malachite green was provided periodically to reload the same concentration of dyes as the initial concentration. For each reactor, 15 batches were performed, and time intervals between 2 successive batches were recorded. Residual malachite green was measured after every batch reaction. Dye decolorization rate *v* was calculated using Equation (2), where *D_0_* is dye concentration at the beginning of each batch, *D_t_* is dye concentration at the end of each batch, and *T* is the time interval between two successive batches.

*v* = *(D_0_ − D_t_)* / *T* (2)

### 3.5. Modeling by Multiple Linear Regression

The molar ratio between CsTMR and BzGDH and the concentrations of substrate and product were treated as independent variables *x_1_*, *x_2_*, and *x_3_*, respectively, and dye decolorization rate was used as the dependent variable (*y*). Multiple linear regression (MLR) was employed to model the relationship between independent variables and dependent variable using the least-squares method by fitting Equation (3) to experimental data,

*y* = *b_0_* + *b_1_x_1_* + *b_2_x_2_* + *b_3_x_3_* (3)

where *b_0_* is the intercept, and *b_1_*, *b_2_*, and *b_3_* are regression coefficients. 

### 3.6. Modeling by Random Forest

The random forest (RF) algorithm proposed by Breiman has been extensively used for classification and regression based on ensembles of a large number of individual decision trees [16]. Each decision tree of the random forest is constructed by using the bootstrap algorithm, a method for random sampling with replacement [24]. In each bootstrap dataset, about 2/3 of the samples are drawn as the training subset (in-bag samples) with random replacement, and about 1/3 of the samples are discarded and treated as “out-of-bag” (OOB) samples [16,25]. The training subset is employed to construct the decision tree and the OOB samples are used for internal validation of the model. The mean square error (MSE, Equation (4)) and the correlation coefficient (R^2^) of the OOB samples are adopted to optimize the number of trees.

*MSE* = Σ(*y_ip_* − *y_ie_*)^2^ / *N* (4)

where *y_ip_* and *y_ie_* are the predicted and experimental values, respectively, and *N* is the number of samples.

Finally, all samples were randomly divided into training dataset and test dataset, and the training dataset was employed to train an RF model using the best tree number. The test dataset was employed to evaluate the generalization capability of the final RF model.

### 3.7. Modeling by Artificial Neural Network

To describe the kinetic behavior of this bienzyme system, a three-layered feed-forward artificial neural network model using the back-propagation algorithm (BP-ANN) was adopted to explore the relationship among enzymes, the concentrations of substrate and product, and dye decolorization rate. Seventy-five data sets obtained from fed-batch trials were used to train a BP-ANN model using MATLAB R2015a (The MathWorks, Inc., Natick, Massachusetts, United States). The datasets were normalized using Equation (5) to generate data in the range of −1.0 to 1.0:

*x_n_* = 2(*x_i_* − *x_min_*) / (*x_max_* − *x_min_*) − 1 (5)

where *x_min_* and *x_max_* are the extrema of variable *x_i_*, respectively.

The number of neurons in the hidden layer of ANN was set at 2-10, and a 10-fold cross-validation technique was applied for determining the best number of hidden neurons, to avoid over-fitting. In this method, the whole datasets are divided into 10 subsets randomly, one subset is discarded, and the network is trained with the residual subsets and then applied for predicting the discarded subsets. The procedure was repeated for the entire datasets. MSE and R^2^ were used to estimate the performance of the trained BP-ANN with different hidden neurons.

Finally, all samples were randomly divided into training dataset and test dataset, and the training dataset was further divided into training (60%), validation (20%), and test subsets (20%) to train a BP-ANN model using the hidden neuron with the best performance in cross-validation. The initial test dataset was employed to evaluate the generalization capability of the final ANN model.

### 3.8. Model Comparision

The prediction accuracy of the constructed models was evaluated using different statistical parameters including *MSE* and R^2^, mean absolute error (*MAE*, Equation (6)), and mean relative error (*MRE*, Equation (7)):

*MAE* = Σ|*y_ip_* − *y_ie_*| / *N* (6)

*MRE* (%) = Σ|(*y_ip_* − *y_ie_*)/*y_ie_*| ∗ 100 / *N* (7)

### 3.9. Neural Interpretation Diagram

The visualization method, the neural interpretation diagram (NID) proposed by Özesmi [26], was adopted to intuitively represent the connection weights among neurons. The relative intensity and directions of connection weight between neurons are represented by line thickness and line shading, respectively.

### 3.10. Estimation of the Importance of Variables

The relative importance of the input variables on the output was estimated by Garson’s algorithm [19] based on the net weight matrix described by Equation (8), which could be simplified to Equation (9) as follows:

*RI_i_* =Σ*_h_* (|*W_ih_W_ho_*| / Σ*_i_*|*W_ih_W_ho_*|) / Σ*_i_*Σ*_h_*(|*W_ih_W_ho_*| /Σ*_i_*|*W_ih_W_ho_*|) (8)

*RI_i_* =Σ*_h_* (|*W_ih_*| / Σ*_i_*|*W_ih_*|) / Σ*_i_*Σ*_h_*(|*W_ih_*| /Σ*_i_*|*W_ih_*|) (9)

where *I_j_* refers to the relative importance of the *j_th_* input variable; and *N_i_*, *N_h_*, and *W_s_* are input neuron number, hidden neuron number, and connection weights, respectively. The subscripts “*i*”, “*h*”, “*o*” and “*l*”, “*m*”, “*n*” are input, hidden, and output layers, and input, hidden, and output neurons, respectively. 

The weight term between hidden and output layers was eliminated in the simplification process from Equation (8) to Equation (9), which could result in misunderstanding the contribution of the input variables to the outputs. To estimate the importance of variables accurately, a modified Garson’s algorithm [20] was employed as Equation (10):

*RI_i_ =*Σ*_h_* (|*W_ih_*| / Σ*_i_*|*W_ih_*| ∗ |*W_ho_*|) / Σ*_i_*Σ*_h_*(|*W_ih_*| /Σ*_i_*|*W_ih_*| ∗ |*W_ho_*|) (10)

Since Garson’s algorithm adopts the absolute values of weights and omitted the opposite directions of weights, Olden et al. proposed the connection weight approach (CWA, Equation (11)) to more precisely estimate the contribution of input to the output, which uses raw hidden-input and hidden-output connection weights, providing the most accurate quantification variable importance over other commonly used approaches [21].

*I_i_ =*Σ*_h_* (|*W_ih_*| * |*W_ho_*|) (11)

### 3.11. Sensitivity Analysis

Sensitivity analysis was performed according to Lek’s algorithm [23], with some modification, to investigate the change of one variable while the other variables were fixed at their 0th, 20th, 40th, 60th, 80th, and 100th percentiles. Lek et al. proposed plotting 12 data points over a given variable range rather than examining its entire range [23]. In this study, the contribution plots were constructed by varying each input variable across 101 data values delimiting 100 equal intervals over its entire range and holding all other variables constant at their 0th, 20th, 40th, 60th, 80th, and 100th percentiles.

## 4. Conclusions

In the present study, a robust cofactor self-sufficient bienzyme biocatalytic system for dye decolorization was successfully constructed. The performance of the decolorization process was also modeled by employing MLR, RF, and ANN algorithms. Evaluation of these models suggested that a three-layered BP-ANN model with six hidden neurons was capable of predicting the dye decolorization efficiency with the best accuracy. Weights analysis of the ANN model showed that the ratio between two enzymes seemed to be the most influential factor, with a relative importance of 54.99% in the decolorization process. The modeling results confirmed that the neural networks could effectively reproduce experimental data and predict the behavior of the decolorization process.

## Figures and Tables

**Table 1:** The apparent decolorization rates of the bienzyme catalytic system.

	Apparent Decolorization Rates (μmol h^−1^)				
	Molar ratio of CsTMR/BzGDH				
	1:10	1:5	1:1	5:1	10:1
Initial	1.65	2.01	1.25	0.37	0.17
Average	0.28	0.35	0.23	0.23	0.17

**Table 2:** Statistical parameters for comparison of different models ^a^.

Parameters	MLR		RF		ANN	
	Train	Test	Train	Test	Train	Test
MSE	0.0511	0.0706	0.0383	0.0419	0.0013	0.0090
MAE	0.1474	0.1708	0.0974	0.1031	0.0270	0.0487
MRE	48.7130	47.7690	32.9363	24.0703	11.1586	13.2349
R^2^	0.5552	0.5725	0.7377	0.7602	0.9867	0.9527

^a^ MLR, multiple linear regression; RF, random forest; ANN, artificial neural network; MSE; mean square error; MAE, mean absolute error; MRE, mean relative error; R^2^, correlation coefficient.

**Table 3:** Weight matrix of neural network ^1^.

W_i_					W_o_	
Neuron	Variable			Bias	Neuron	Weight
	Ratio	Substrate	Product			
1	−1.0584	−5.3016	−0.5183	6.9997	1	0.0944
2	−5.3136	−32.2206	−3.3973	−17.0822	2	0.1495
3	−2.0184	1.6110	−5.7296	1.1000	3	0.0555
4	−0.7781	−4.5670	13.1172	7.7110	4	−0.3755
5	−0.7376	−5.0937	−0.4546	−5.3149	5	0.6968
6	2.2761	1.3407	0.1931	5.0204	6	12.8454
					**Bias**	−12.5429

^1^ W_i_: weights between input and hidden layers; W_o_: weights between hidden and output layers.

**Table 4:** Importance of input variables on the output layer.

Variables	Importance		
	Garoson ^1^ (%)	Garson_mod_^2^ (%)	CWA ^3^
Ratio	20.94	54.99	28.01
Substrate	52.33	37.83	10.16
Product	26.73	7.19	-3.64

^1^ Garson’s algorithm [19]. ^2^ Modified Garson’s algorithm [20]. ^3^ Connection weight approach [21].
